# Computational reconstruction of proteome-wide protein interaction networks between HTLV retroviruses and Homo sapiens

**DOI:** 10.1186/1471-2105-15-245

**Published:** 2014-07-18

**Authors:** Suyu Mei, Hao Zhu

**Affiliations:** Bioinformatics Section, School of Basic Medical Sciences, Southern Medical University, Guangzhou, 510515 China; Software College, Shenyang Normal University, Shenyang, 110034 China

## Abstract

**Background:**

Human T-cell leukemia viruses (HTLV) tend to induce some fatal human diseases like Adult T-cell Leukemia (ATL) by targeting human T lymphocytes. To indentify the protein-protein interactions (PPI) between HTLV viruses and Homo sapiens is one of the significant approaches to reveal the underlying mechanism of HTLV infection and host defence. At present, as biological experiments are labor-intensive and expensive, the identified part of the HTLV-human PPI networks is rather small. Although recent years have witnessed much progress in computational modeling for reconstructing pathogen-host PPI networks, *data scarcity* and *data unavailability* are two major challenges to be effectively addressed. To our knowledge, no computational method for proteome-wide HTLV-human PPI networks reconstruction has been reported.

**Results:**

In this work we develop Multi-instance Adaboost method to conduct homolog knowledge transfer for computationally reconstructing proteome-wide HTLV-human PPI networks. In this method, the homolog knowledge in the form of *gene ontology* (*GO*) is treated as auxiliary *homolog instance* to address the problems of *data scarcity* and *data unavailability*, while the potential *negative knowledge transfer* is automatically attenuated by AdaBoost instance reweighting. The cross validation experiments show that the homolog knowledge transfer in the form of independent *homolog instances* can effectively enrich the feature information and substitute for the missing *GO* information. Moreover, the independent tests show that the method can validate 70.3% of the recently curated interactions, significantly exceeding the 2.1% recognition rate by the HT-Y2H experiment. We have used the method to reconstruct the proteome-wide HTLV-human PPI networks and further conducted *gene ontology* based clustering of the predicted networks for further biomedical research. The *gene ontology* based clustering analysis of the predictions provides much biological insight into the pathogenesis of HTLV retroviruses.

**Conclusions:**

The Multi-instance AdaBoost method can effectively address the problems of *data scarcity* and *data unavailability* for the proteome-wide HTLV-human PPI interaction networks reconstruction. The *gene ontology* based clustering analysis of the predictions reveals some important signaling pathways and biological modules that HTLV retroviruses are likely to target.

**Electronic supplementary material:**

The online version of this article (doi:10.1186/1471-2105-15-245) contains supplementary material, which is available to authorized users.

## Background

Pathogen-host protein-protein interactions (PPI) play important roles in the process of pathogen infection and host response. Fast and accurate mapping of proteome-wide pathogen-host protein interactome provides valuable insight into the underlying pathogenesis of pathogens and promotes discovery of novel druggable targets. As compared with labor-extensive and expensive experimental methods, computational methods facilitate the fast reconstruction of proteome-wide pathogen-host PPI networks at low cost. At present, most computational methods focus on *intra-species* PPI network reconstruction (e.g. yeast PPI network [1], *Arabidopsis thaliana* PPI network [2], human PPI network [3], etc.) in that the experimentally-derived *intra-specie*s PPI networks are large enough for computational modeling, though with noise and far from complete [4, 5]. In contrast, the host-pathogen PPI networks available are still very small. For instances, the latest HIV-human PPI database [6] contains 3,638 interactions, the *P.falciparum*-*H.sapiens* PPI dataset [7] contains 1,112 interactions, and the smallest *Salmonella*-human PPI dataset [8] contains just 62 interactions. Schleker et al. [9] used HT-Y2H (high-throughput yeast-two-hybrid) to detect 166 interactions between HTLV (Human T-cell lymphotropic viruses) and human proteins. Such small pathogen-host PPI datasets are prone to yield model overfitting.

Most of the reported computational methods for pathogen-host PPI prediction focus on the pathogens like HIV-1 [10–14], *P.falciparum* [15], *Salmonella* [16–18], etc., and generally leverage multiple biological feature information as shown in Table 1. Integration of feature information truly improves the model performance to a certain degree, but it has the two major demerits: (1) aggregation of multiple feature information without augmenting the training data is prone to cause model overfitting on small training data; (2) integration of feature information poses demanding data constraints on the computational modeling. When the feature information is not available to test data, the trained model will fail to work. Thus, how to effectively substitute for the potentially missing feature information is a major issue of computational modeling. In [17, 19], the missing feature information such as *gene ontology* (*GO*) and *gene expression* was elaborately substituted with the homolog *GO* knowledge and protein sequences. The feature information of protein sequences, though cheap to obtain, is criticized for its poor predictive power [20].

**Table 1:** **Summary of feature information extracted from literatures**

***Integration of feature information***	***Literatures***
*Sequence k-mer*, *interlog*, *gene ontology*, *metabolic pathways*	[7]
*Binding motif*, *gene expression profile*, *gene ontology*, *sequence similarity*, *post-translational modification*, *tissue distribution*, *PPI network topology*	[10, 11]
*Protein domain profile*, *sequence k-mer*	[12]
*Structural similarity*	[13]
*Protein domain profile*, *gene expression*, *gene ontology, gene co-expression*	[15]

As a member of the family of retroviruses, Human T-cell lymphotropic viruses (HTLV) are divided into two sub-types. The type 1 virus (HTLV-1) is known to induce Adult T-cell Leukemia/Lymphoma (ATL), but what diseases are caused by the type 2 virus (HTLV-2) remain unclear [9]. The HT-Y2H (high-throughput yeast-two-hybrid) [21, 22] was used to yield 166 interactions between HTLV and human proteins. However, this HT-Y2H study validated only three interactions between HTLV-1 Tax and three human proteins (Nup62, MAD1L1, Cdc23) that have been collected in the databases *VirusMINT* [23] and *VirHostNet* [24]. Since there are 145 HTLV-human PPIs in the two databases, this HT-Y2H study achieves only 2.1% recognition rate of experimentally derived PPIs. Such a low recognition rate is partly caused by different sensitivity of experimental methods to different types of interaction. Computational modeling can shield the low-level biochemical specificity (e.g. covalent modification) of protein-protein interactions to set up a general-purpose PPI predictor. To our knowledge, no computational method has been developed for fast reconstruction of proteome-wide HTLV-human PPI networks.

In this work, we propose a computational method that addresses the problems of *data unavailability* and *data scarcity* for reconstructing proteome-wide HTLV-human PPI networks. The homolog knowledge, in terms of *gene ontology* (*GO*), is treated as auxiliary *homolog instances* to mingle with the *target instances* (the *GO* knowledge of the proteins themselves), such that (1) the *homolog instances* augment the training data to reduce the risk of model overfitting; (2) the feature information is enriched to make up for *data scarcity*; (3) the *homolog instances* are used as substitute when the *target instances* are not available. It is noted that such a way of homolog knowledge transfer may introduce a certain level of noise that results from evolutionary divergence. On the basis of the original instance reweighting AdaBoost [25, 26], we propose Multi-instance Adaboost to attenuate the noise from *homolog instances*. The model performance is evaluated by 10-fold cross validation and independent test. Last, we use Multi-instance AdaBoost to reconstruct the proteome-wide HTLV-human PPI networks and further conduct *gene ontology* based clustering analysis of the predictions to gain insight into the pathogenesis of HTLV retroviruses.

## Methods

### Data and materials

The training data are collected from two sources, one dataset is from [9] that contains 166 interactions (hereinafter called *S*1_*pos*_), and the other dataset is from the two databases [23, 24] that contains 145 interactions (hereinafter called *S*2_*pos*_). After removing those putative/uncharacterized/uncurated/hypothetic HTLV proteins and those HTLV proteins that have no corresponding accessions in the Uniprot database (http://www.uniprot.org/uniprot/), *S*1_*pos*_ is reduced to 155 interactions between 9 HTLV proteins and 112 human proteins. Accordingly, the negative data of equal size are randomly sampled for *S*1_*pos*_ and, called *S*1_*neg*_, *S*2_*neg*_, respectively. Then the two training data are defined as *S*1 = *S*1_*pos*_ ∪ *S*1_*neg*_ and *S*2 = *S*2_*pos*_ ∪ *S*2_*neg*_, and the whole training data is defined as *S* = *S*1 ∪ *S*2. It is noted that each training data are actually doubled in size, because each data point is represented with two instances, i.e., the *target instance* and the *homolog instance*.

### GO feature construction

The homologs are extracted from SwissProt database [27] using PSI-BLAST [28] (*E-value* = 10) and the *gene ontology* (*GO*) terms are extracted from GOA database [29]. To increase the coverage of homologs, we adopt default *E-value* (*E-value* = 10) of PSI-BLAST and search for the space of all the species available in *SwissProt* database. For each protein *i,* we obtain two sets of *GO* terms, one set contains the *GO* terms from the homologs denoted as *homolog set*, and the other set contains the *GO* terms from the protein itself denoted as *target set*. Based on the denotations, we can formally define two feature vectors for a protein pair (*i*_1_, *i*_2_) as follows:
1

where  denotes component *g* of the *target instance* and  denotes component *g* of the *homolog instance*. In practical implementation, each *GO* term *g* is assigned an integer index. Formula (1) means that if the protein pair (*i*_1_, *i*_2_) shares the same *GO* term *g*, then the corresponding component in the feature vector  or  is set 2; if neither protein in the protein pair possesses the *GO* term *g,* then the value is set 0; otherwise the value is set 1. The above definition is symmetrical, i.e., the protein pair (*i*_1_, *i*_2_) and the protein pair (*i*_2_, *i*_1_) have identical feature representation.

### Multi-instance AdaBoost

In the scenario of traditional machine learning, data point is generally represented with only one instance, whereas only one instance is not enough to depict a biological molecule (e.g. protein, DNA, RNA) in computational studies. For instance, a series of multi-aspect information is needed to depict the temporal and spatial information of DNA transcription, protein folding, etc. Moreover, evolutionary information may be needed to provide abundant knowledge about the biological molecule concerned. To address the problem, we are motivated to explore multi-instance learning to enrich protein information by representing proteins with more than one instance.

Here we depict each protein with two instances, one instance called *target instance* is used to represent the *gene ontology* (*GO*) information of the protein itself, and the other instance called *homolog instance* is used to represent the *GO* information of the homologs. The *homolog instance* is used to capture the evolutionary information as well as to enrich the feature information of the *target instance*. Meanwhile, the *homolog instance* also plays an important role in tackling the problem of data unavailability. When the feature information indispensible for prediction is not available, the *homolog instance* can be treated as substitute for the *target instance* to guarantee that the model still works. However, in some cases the *homolog instances* are likely to carry noise because of evolutionary divergence, thus it is not proper to treat the two kinds of instances equally. One way to solve the problem is to assign different weight distributions to the two kinds of instances, so that the predictive model can be boosted to generalize well. To our knowledge, AdaBoost [25, 26] is a boosted ensemble classifier that iteratively reweight the instances according to the difficulty of classification. AdaBoost instance reweighting [25, 26] is defined as follows:
2

where *x*_*i*_ denotes the *i*-th training instance, *y*_*i*_ denotes its class label, *f*_*m*_(*x*_*i*_) denotes the decision value predicted by the committed obtained in the *m*-th round of training, *D*_*m*_(*i*) denotes the weight of the *i*-th training instance in the *m*-th round of training, and *Z*_*m*_ denotes the normalizer. From Formula (1), we can see that AdaBoost assigns high weights to those hard-to-classify instances and assigns low weights to those easy-to-classify instances for the next round of training. This idea of iterative reweighting of the training samples is essential to Boosting. Intuitively speaking, the examples that are misclassified get higher weights in the next iteration, for instance, the noisy/outlier examples near the decision boundary are usually harder to classify and therefore get high weights after a few iterations [30]. In [30], it has been theoretically proven that the boosted ensemble classifier achieves a large margin between two-class hyperplanes through multiple rounds of instances reweighting. From a theoretical point of view, AdaBoost implicitly penalizes the *ℓ*_1_ norm [27], and the regularization technique penalizes the impact of noise/outlier at the cost of higher training error to achieve lower generalization error.

The latest AdaBoost (Modest AdaBoost, [26]) combines the distribution of instance weights and its inverted distribution into a decision function to make the decision “soft” (see Additional file 1). Based on Modest AdaBoost, we develop the Multi-instance AdaBoost method to conduct *homolog knowledge transfer*. As compared to single-instance AdaBoost, Multi-instance AdaBoost shows no much difference in the training phase, except that each protein pair is represented by two instances  and  as defined in Formula (1). The mail difference lies in the test phase, where the decision committee  yields two outputs  for any test pair (*i*_1_, *i*_2_) ( is the decision function of Modest AdaBoost, see Additional file 1). The final decision value for (*i*_1_, *i*_2_) is defined as below:

3

where | • | denotes absolute value. Then the final label for (*i*_1_, *i*_2_) is defined as below:
4

### Model evaluation

We design three experimental settings to validate the effectiveness of the proposed Multi-instance Adaboost. The first setting is Single-instance AdaBoost, used as the baseline model to evaluate the performance gain from *homolog instances*. In this setting, each protein pair is represented by the *target instance*, without introducing *homolog instance*. The second setting is Multi-instance AdaBoost Novel, deliberately designed to evaluate the model robustness to data unavailability. In this setting, the training data are represented by two kinds of instances, while the test data are represented with *homolog instances* alone to simulate data unavailability. The third setting is Multi-instance AdaBoost, designed to evaluate the model capability of overcoming data scarcity. In this setting, both the training data and the test data are represented by the two kinds of instances.

We estimate the model performance for the three settings using 10-fold cross validation and independent test. *Receiver Operating Characteristic* (*ROC) AUC* (*Area Under Curve*) (referred to as *ROC-AUC*), *Precision recall curve AUC* (*PR-AUC*), *Specificity* (*SP*), *Sensitivity* (*SE*), *MCC* (*Matthews correlation coefficient*), *F1 score* and *Overall Accuracy* are adopted as performance metrics. The formal definitions of the performance metrics are given in the Additional file 1.

## Results and discussion

### Model performance evaluation

Before proteome-wide predictions, we first evaluate the reliability of Multi-instance AdaBoost. In [9], the experimental HT-Y2H recognized 166 interactions and validated only three interactions out of the 145 interactions collected in the two databases [23, 24]. Of the two datasets, the former dataset [9] is processed and named as *S*1_*pos*_ in this work, and the latter dataset [23, 24] is named as *S*2_*pos*_. Through random sampling we obtain the corresponding negative datasets *S*1_*neg*_, *S*2_*neg*_ for the two positive datasets *S*1_*pos*_ and *S*2_*pos*_, respectively. Thus we obtain two training datasets: *S*1 = *S*1_*pos*_ ∪ *S*1_*neg*_ and *S*2 = *S*2_*pos*_ ∪ *S*2_*neg*_. To our knowledge, there is no existing computational method for HTLV-human PPI prediction, so we use the HT-Y2H recognition rate of novel PPIs [9] as the baseline performance. To compare with HT-Y2H, we use *S*1 to train Multi-instance AdaBoost and then check how many interactions out of *S*2_*pos*_ can be correctly recognized. This evaluation is actually an independent test, i.e., *S*2_*pos*_ is used as an independent test set to validate the model that is trained on *S*1. Before validating *S*2_*pos*_, we conduct 10-fold cross validation model evaluation on the training data *S*1.

#### 10-fold cross validation model evaluation

**Figure 1:**
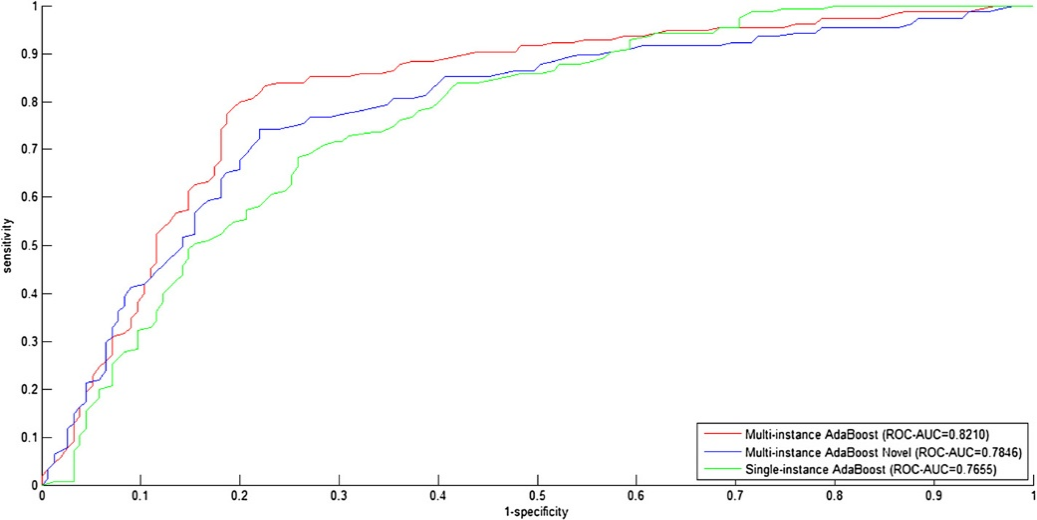
**ROC curves for three experimental settings (Multi-instance AdaBoost, Multi-instance AdaBoost Novel, Single-instance AdaBoost) on the dataset** ***S1.***

**Figure 2:**
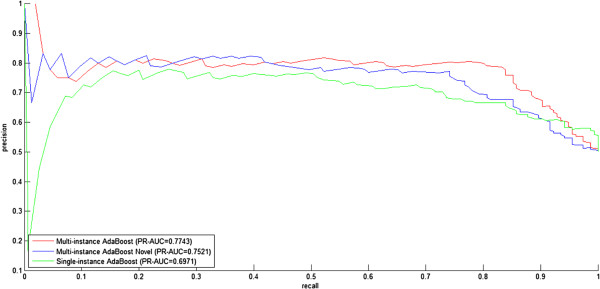
**Precision-recall curves for three experimental settings (Multi-instance AdaBoost, Multi-instance AdaBoost Novel, Single-instance AdaBoost) on the dataset** ***S1.***

The results of 10-fold cross validation for the three settings on dataset *S1* are illustrated in Figures 1, 2 and Table 2. We use the setting Single-instance AdaBoost as the baseline to demonstrate the effectiveness of homolog knowledge transfer by means of independent homolog instances.

**Table 2:** **10-fold cross validation performance estimation on the dataset** ***S1***

	***Multi-instance AdaBoost***			***Multi-instance AdaBoost Novel***			***Single-instance AdaBoost***		
	***SP***	***SE***	***MCC***	***SP***	***SE***	***MCC***	***SP***	***SE***	***MCC***
***Positive (interaction)***	0.7647	0.8387	0.6498	0.7011	0.7871	0.5611	0.6784	0.7484	0.5192
***Negative (non-interaction)***	0.8214	0.7419	0.6397	0.7574	0.6645	0.5416	0.7194	0.6452	0.5001
**[** ***Acc; MCC*** **]**	[79.03%; 0.6426]			[72.58%; 0.5487]			[69.58%;0.5080]		
**[** ***ROC*** **-** ***AUC; PR*** **-** ***AUC*** **]**	[ 0.8210; 0.7743]			[ 0.7846; 0.7521]			[ 0.7655; 0.6971]		
***F1 score***	0.80			0.74			0.71		

**Multi-instance AdaBoost versus single-instance AdaBoost**. From Figures 1 and 2, we find that Multi-instance AdaBoost significantly outperforms the baseline setting Single-instance AdaBoost, with *ROC-AUC* 0.8210 versus 0.7655 and *PR-AUC* 0.7743 versus 0.6971, respectively. From Table 2, we also find that that Multi-instance AdaBoost shows significantly better performance than Single-instance AdaBoost with *overall Accuracy* 79.03% versus 69.58%. The results suggest that the *homolog instances* are effective to enrich the feature information and solve the problem of data scarcity. Further details in Table 2 provide additional information about the predictions. For the three settings, the recall rates (sensitivity, *SE*) of the positive class (interaction) are generally higher than those of the negative class (non-interaction), and conversely the specificity (*SP*) values of the positive class (interaction) are generally lower than those of the negative class (non-interaction), suggesting that the negative class yields larger misclassification rate than the positive class. To reduce the misclassification rate, we need improve the quality of the sampled negative data. At present, there is no experimentally derived golden-standard non-interaction data, and random sampling is often used as an alternative to obtain the negative data. As we know, random sampling is prone to sample false negative data and thus introduce a certain level of noise. How to sample quality negative data deserves our future study. In this study, random sampling seems to introduce no obvious predictive bias in the three settings from the points of view of the *MCC* values on the positive class and the negative class, e.g., Multi-instance AdaBoost (0.6498, 0.6397), Multi-instance AdaBoost Novel (0.5611, 0.5416) and Single-instance AdaBoost (0.5192, 0.5001).

**Multi-instance AdaBoost novel versus single-instance AdaBoost**. From Figures 1 and 2, we find that Multi-instance AdaBoost Novel still outperforms the baseline setting Single-instance AdaBoost, with *ROC-AUC* 0.7846 versus 0.7655 and *PR-AUC* 0.7521 versus 0.6971, respectively. From Table 2, Multi-instance AdaBoost also shows better performance than Single-instance AdaBoost with *overall Accuracy* 72.58% versus 69.58%. The results, though not so significant as Multi-instance AdaBoost, still suggest that the *homolog instances* are effective to substitute for the *target instances* and thus securely avoid model failure when the *gene ontology* knowledge is not available.

**Multi-instance AdaBoost versus other pathogen-host PPI predictors**. We can not conduct direct model comparison because no computational model has been developed for HTLV-human PPI prediction thus far. For rough knowledge about the reliability of Multi-instance AdaBoost, we conduct indirect comparisons with two representative pathogen-host PPI predictive models. One model is the semi-supervised multi-task learning method for HIV-human PPI prediction [11] and the other model is the random forest for *Salmonella-*human PPI prediction [17]. The model for HIV-human PPI prediction is trained on large data (2,277 interactions) and achieves 0.919 *ROC-AUC* score, whereas the model for *Salmonella-*human PPI prediction is trained on rather small data that contains only 66 interactions and achieves 0.52 *F1* score. We can see that the size of training data is one of the factors that have large influence on the model performance. Comparatively, the proposed Multi-instance AdaBoost achieves 0.8210 *ROC-AUC* score and 0.80 *F1* score. In terms of training data size, the Multi-instance AdaBoost model trained on 155 interactions is much closer to the *Salmonella-*human PPI prediction model (66 interactions) than to the HIV-human PPI prediction model (2,277 interactions). Nevertheless, Multi-instance AdaBoost achieves a significantly higher *F1* score than the *Salmonella-*human PPI prediction model (0.80 versus 0.52). Moreover, Multi-instance AdaBoost achieves at least 0.7419 *SE* on the positive class, also significantly higher than the *Salmonella-*human PPI prediction model (*SE* 0.407). These rough comparisons, though based on different data, suggest that the proposed Multi-instance AdaBoost performs well on small data.

#### Independent test on the data from recent databases

As mentioned above, the experimental HT-Y2H [9] reproduced only three interactions out of the 145 interactions collected from *VirusMINT* [23] and *VirHostNet* [24], accounting for 2.1% *recognition rate*. The result suggests that HT-Y2H is effective to some specific protein-protein interactions (e.g. transient interaction) but is prone to yield rather high false negative rate for other types of interaction. Furthermore, not only is the overlap between different experimental results rather small, but also the overlap between the computationally reconstructed network and the experimentally derived network is neither large. As reported in [11], the semi-supervised multi-task learning model validated only 10% HIV-human PPIs derived by *siRNA screen*. The low network overlap may suggest two points: (1) different experimental techniques should be treated as mutual complements to detect different types of protein-protein interaction, or (2) the computational methods need further improvement to generalize well.

The results of 10-fold cross validation shows that the proposed Multi-instance AdaBoost achieves better performance on small data than other existing pathogen-host PPI predictor [17]. Here we further conduct an independent test to compare with the experimental HT-Y2H [9] by examining how many interactions out of the 145 interactions (*S*2_*pos*_) can be correctly recognized by Multi-instance AdaBoost. The independent test is actually a validation on the positive data *S*2_*pos*_ with negligence of the negative data *S*2_*neg*_, as we are more concerned about the recognition rate of the known PPIs. For this reason, we train Multi-instance AdaBoost on the dataset *S1* and use the model to predict *S*2_*pos*_. Notably, Multi-instance AdaBoost can correctly recognize 102 interactions out of the total 145 interactions (*S*2_*pos*_), accounting for 70.3% *recognition rate*, much larger than HT-Y2H 2.1% *recognition rate* [9] and 10% overlap between predictions and *siRNA screen* [11]. The overlap between the networks predicted by Multi-instance AdaBoost and derived by HT-Y2H is given in Additional file 2.

### Proteome-wide PPIs prediction and gene ontology based clustering analysis

#### Proteome-wide PPIs prediction

In this section we exploit the PPI data available [9, 23, 24] to train Multi-instance AdaBoost for proteome-wide HTLV-human PPI networks reconstruction. Before predictions, we also conduct 10-fold cross validation model evaluation on the whole dataset *S*. The results are equivalent to the 10-fold cross validation performance on the dataset *S1* (see Additional file 1: Figure S1, Figure S2 and Table S1).

In the dataset *S*, there are 9 HTLV proteins that have corresponding reviewed accessions in the Uniprot database. The human proteins are taken from the file *uniprot_sprot_human.dat.gz* available at ftp://ftp.uniprot.org/pub/databases/uniprot/ current_release/knowledgebase/taxonomic_divisions/. After removing those uncurated/putative/uncharacterized proteins and those proteins that are already used as training data, we finally obtain 20,334 human proteins as the candidate targets of the 9 HTLV proteins. Hence there are totally 183,006 (9 × 20,334) protein pairs to be predicted. We use the trained Multi-instance AdaBoost to predict all the 183,006 protein pairs and detect *61,846* novel interactions (see Additional files 2 and 3), accounting for 33.79% *predicted positive rate*. Among the 20,334 human proteins, there are totally 10,445 human proteins predicted to interact with the 9 HTLV proteins, that’s to say, about 50% of the known human proteins are predicted to be potentially targeted by HTLV proteins. The result suggests that the proposed Multi-instance AdaBoost yields a certain degree of false positive predictions. The risk of false positive is a hard problem to both computational modeling and high-throughput biological experiments [11]. The problem seems to be more serious when the training data is very small. The *Salmonella-*human PPI predictor [17] set the decision probability threshold at 0.7 and predicted 22,651 human proteins out of 22,654 human proteins to interact with 25 *Salmonella* proteins. The percentage of interacting human partners is up to 99.99%, suggesting a much higher risk of overprediction than the proposed Multi-instance AdaBoost. Comparatively, Multi-instance AdaBoost is much more reliable than the *Salmonella-*human PPI predictor [17] in terms of false positive rate. If we further add a threshold of decision value to Formula (4), i.e., |*Decision* _ *value*(*i*_1_, *i*_2_)| > *δ*, the risk of false positive predictions would be greatly reduced. The threshold *δ* is at the discretion of users for choosing reliable predicted interactions. Through comparison with the existing pathogen-host PPI predictors, the proposed Multi-instance AdaBoost, though yielding a certain degree of false positive predictions, is reliable to reconstruct the proteome-wide HTLV-PPI networks valuable for biological research and can be used as baseline model for further computational modeling.

#### Gene ontology based clustering analysis

In this section, we further study the predicted interactions to gain biological insight into the general patterns that HTLV viruses attack human proteins. We simply cluster together the HTLV targeted human proteins that fulfil identical molecular functions, participate in the same biological processes, collaborate within the same signaling pathways or reside in the same cellular compartments. Thus each cluster defines a biological module*,* within which all the human proteins share a specific biological character. As regards with clustering algorithm, how to define the biometric distance is an important concern. Here we use *gene ontology* term (*GO term*) as distance metric, i.e., the interacting human partners that possess the same *GO term* are assigned to the same cluster. Thus each *GO* term corresponds to a cluster or biological module.

All the *GO terms* of human proteins are classified into thee major classes, i.e., *biological processes* (P), *molecular functions* (F) and *cellular compartments* (C). For each major class, we further discuss the two cases: (1) all the 9 HTLV proteins are involved in the biological module*,* denoted as P1, F1 and C1, respectively; (2) NOT all the 9 HTLV proteins are involved in the biological module*,* denoted as P2, F2 and C2, respectively. P1, F1 and C1 are given in Additional files 4, 5 and 6, respectively. P2, F2 and C2 are given in Additional files 7, 8 and 9, respectively. For the sake of large number of biological modules (clusters), we only demonstrate two biological modules here as examples, interested readers are referred to Additional files 4, 5, 6, 7, 8 and 9 for other biological cues.

**Figure 3:**
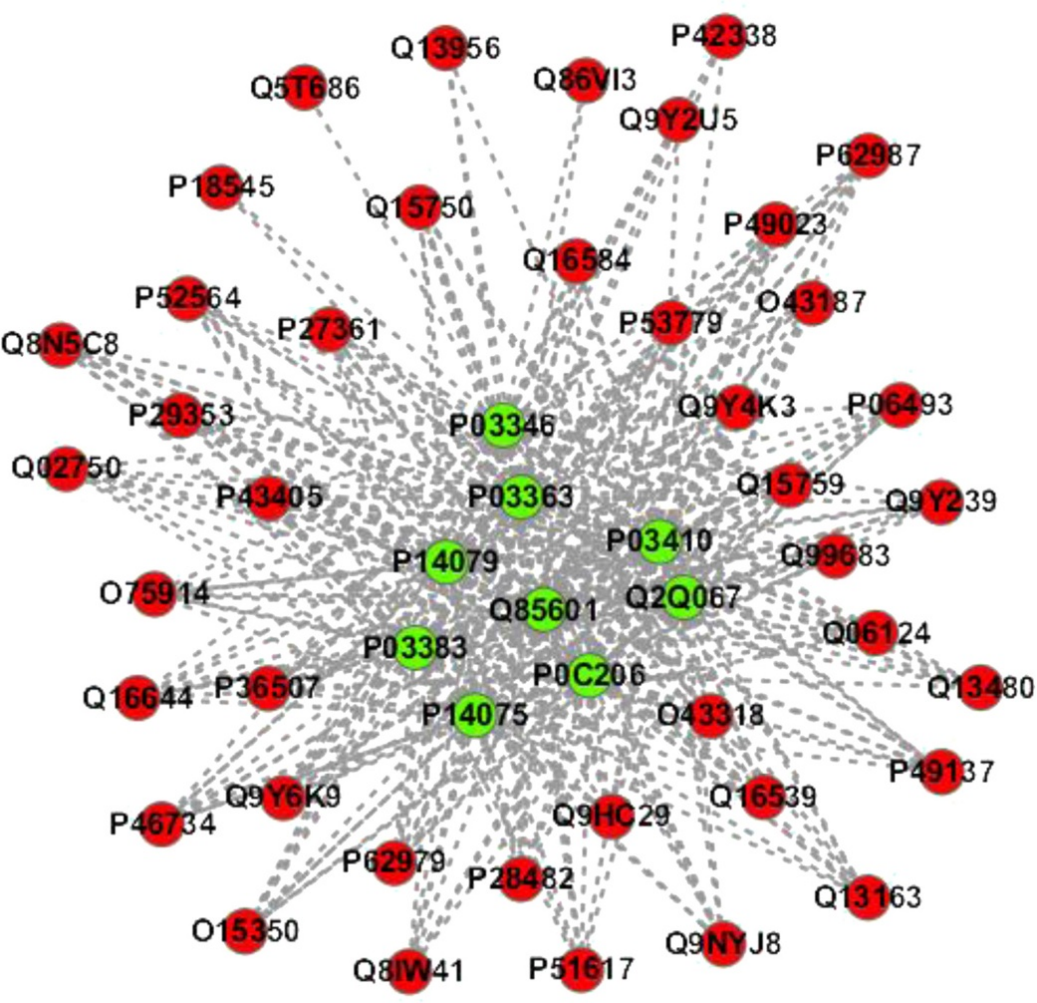
**The predicted HTLV-human PPI sub-network GO:0000187 (** ***biological process: activation of MAPK activity*** **).** The green node denotes HTLV protein and the red node denotes human protein.

**PPI sub-network GO:0000187 - activation of MAPK activity**. The predicted PPI sub-network GO:0000187 is extracted from Additional file 4 and illustrated in Figure 3. As shown in Figure 3, the 9 HTLV proteins are predicted to interact with the human proteins that are involved in the biological processes “*activation of MAPK activity*” (GO:0000187). In the predicted PPI sub-network, some human proteins are predicted to be targeted by all the 9 HTLV proteins (e.g. P49023, P49137, Q8N5C8, P28482, Q9Y4K3, O75914, Q15759, P62979, etc.). two proteins (P18545, Q5T686) are predicted to interact with only one HTLV protein, and the other human proteins are predicted to interact with 2 ~ 8 HTLV proteins. From the definition of GO:0000187- *the initiation of the activity of the inactive enzyme MAP kinase by phosphorylation by a MAPKK*, we can infer that the 9 HTLV proteins are likely to interfere with host MAPK signaling pathways.

**Figure 4:**
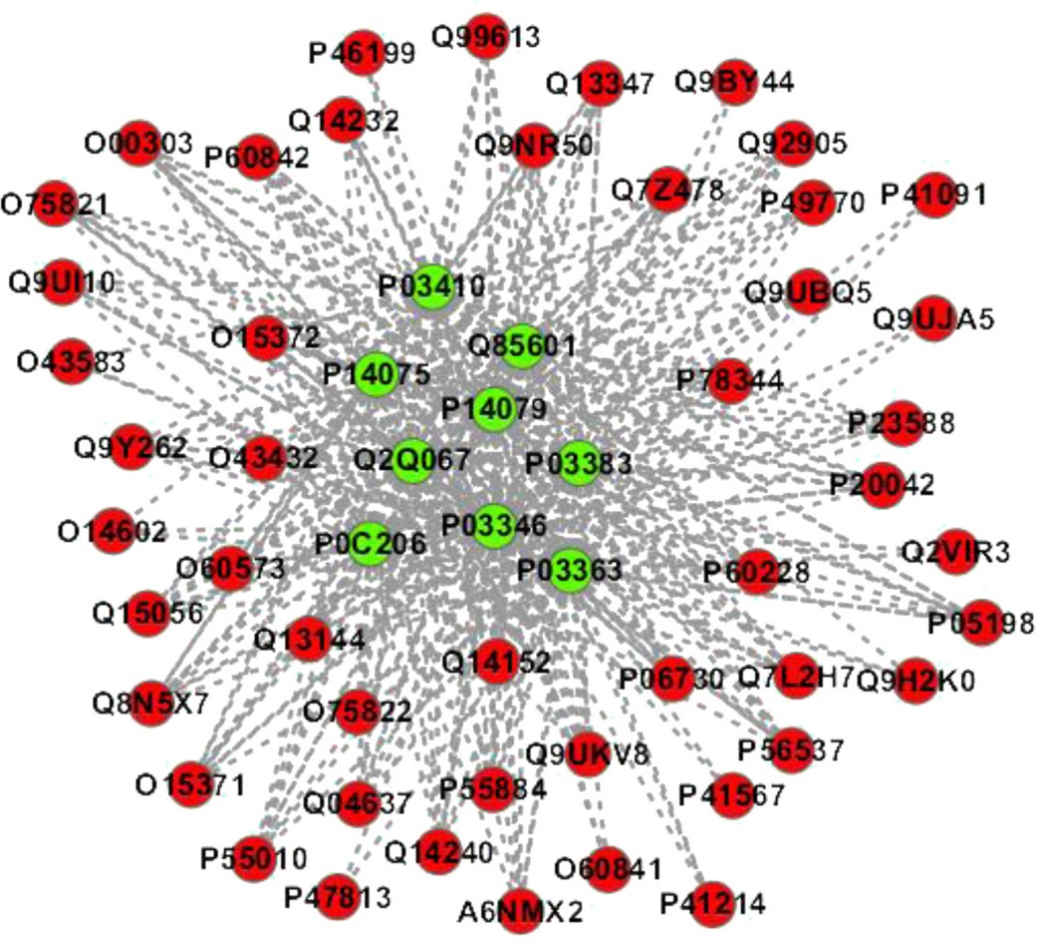
**The predicted HTLV-human PPI sub-network GO:0003743 (** ***molecular function: translation initiation factor activity*** **).** The green node denotes HTLV protein and the red node denotes human protein.

**PPI sub-network GO:0003743 - translation initiation factor activity**. The predicted PPI sub-network GO:0003743 is extracted from Additional file 5 and illustrated in Figure 4. From Figure 4, we can see that the human partners within the predicted PPI sub-network generally interact with multiple HTLV proteins that fulfil the molecular function “*translation initiation factor activity*” (GO:0003743). According to the definition of GO:0003743-*functions in the initiation of ribosome-mediated translation of mRNA into a polypeptide*, we can infer that the 9 HTLV proteins are likely to interfere with host mRNA translation.

It is noted that not all the 9 HTLV proteins are necessarily involved in the same biological module (cluster). We also extract the PPI sub-network GO:0000187 from Additional file 7 (see Additional file 1: Figure S3) and PPI sub-network GO:0003743 from Additional file 8 (see Additional file 1: Figure S4) as illustrative examples.

## Discussion

Human T-cell lymphotropic virus (HTLV-1) is a known retrovirus that can induce *Adult T-cell Leukemia* (ATL) and *Tropical Spastic Paraparesis* (TSP). At present, the HTLV-human PPI networks are so small to limit our knowledge about the underlying mechanism of HTLV infection and human response. The small overlap between networks derived by different experimental techniques suggests that each experimental technique has strong specificity to specific types of protein-protein interaction. Computational modeling is a good complement to highly specific experimental methods to fast and cheaply reconstruct the proteome-wide HTLV-human PPI networks. However, computational modeling on small data is prone to model overfitting. How to overcome the bottleneck of small training data is the major concern of computational modeling.

In this work, we propose Multi-instance AdaBoost to address the problems of *data scarcity* and *data unavailability* for proteome-wide HTLV-human PPI networks reconstruction. In this method, the *gene ontology* knowledge of the homologs is treated as independent *homolog instance* to augment the training data, so that the feature information is enriched to make up for *data scarcity* and reduce the risk of model overfitting. Meanwhile, the *homolog instances* are treated as substitute for the potentially missing *target instances* to address the problem of *data unavailability*. However, since the *homolog instances* are likely to carry a certain level of noise due to evolutionary divergence, we resort to AdaBoost instance reweighting to attenuate the impact of noise. AdaBoost has been theoretically proven to maximize the margin between two-class hyperplanes by penalizing the impact of noise/outlier. As compared to other existing pathogen-host PPI predictive models [17, 18], the proposed Multi-instance AdaBoost has several advantages: (1) the homolog knowledge is used to augment the training data and thus to reduce the risk of model overfitting; (2) the homolog knowledge is used as substitute to address the problem of data unavailability; (3) the noise from homolog knowledge transfer is attenuated by AdaBoost instance reweighting algorithm. Comparatively, a drawback of Multi-instance AdaBoost is that the other feature information except *gene ontology* is not integrated into the model. We should achieve balance between data constraint and data enrichment in the future research.

To validate the assumptions that the *homolog instances* are effective to address the problems of *data scarcity* and *data unavailability*, we design three experimental settings, i.e. Multi-instance AdaBoost, Multi-instance AdaBoost Novel and Single-instance AdaBoost, and conduct 10-fold cross validation experiments & independent tests for each setting, using multiple performance metrics (*SP, SE, Accuracy, MCC, ROC-AUC*, *PR-AUC*). The experimental results demonstrate these points: (1) Multi-instance AdaBoost significantly outperforms the baseline Single-instance AdaBoost, suggesting that the *homolog instances* are effective to augment the training data; (2) Multi-instance AdaBoost Novel still outperforms the baseline Single-instance AdaBoost, suggesting that the proposed Multi-instance AdaBoost can still work well when the feature information of the proteins to be predicted is not available; (3) Multi-instance AdaBoost correctly recognize 70.3% of the known PPIs, significantly higher than HT-Y2H 2.1% recognition rate; (4) Indirect comparisons show that Multi-instance AdaBoost outperforms the existing pathogen-host PPI predictive models that were trained on small datasets.

Lastly, we apply Multi-instance AdaBoost to reconstruct the proteome-wide HTLV-human PPI networks and conduct *gene ontology* based clustering analysis of the predicted networks. The clustering analysis gains much insight into the pathogenesis of HTLV retroviruses and provides valuable clues for further experimental studies.

## Conclusion

The computational modeling for pathogen-host PPI networks reconstruction needs to address the major concerns of *data scarcity* and *data unavailability*. In this paper, we propose a novel method Multi-instance AdaBoost to augment the training data. Experimental results show that the homolog knowledge transfer by means of independent *homolog instances* is effective to enrich the information abundance and to help the model work properly when the feature information is not available. Moreover, the *gene ontology* based clustering of the proteome-wide predicted HTLV-human PPI networks provides valuable clues for further biomedical research.

## Electronic supplementary material

Additional file 1: **Brief description of Modest AdaBoost [** [26]**].** Formal definitions of SP, SE, MCC, overall accuracy (Acc) and F1 score. **Figure S1** ROC curve for three experimental settings (Multi-instance AdaBoost, Multi-instance AdaBoost Novel, Single-instance AdaBoost) on the dataset *S*. **Figure S2** Precision-Recall curve for three experimental settings (Multi-instance AdaBoost, Multi-instance AdaBoost Novel, Single-instance AdaBoost) on the dataset *S*. **Table S1** 10-fold cross validation performance estimation on on the dataset *S.* **Figure S3** The predicted HTLV-human PPI sub-network GO:0006120 *(biological process: mitochondrial electron transport, NADH to ubiquinone*). The green node denotes HTLV protein and the red node denotes human protein. **Figure S4** The predicted HTLV-human PPI sub-network GO:0005344 (*molecular function: oxygen transporter activity*). The green node denotes HTLV protein and the red node denotes human protein. (PDF 139 KB)

Additional file 2: **Text file contains the overlapped interactions between Multi-instance AdaBoost and the experimental technique HT-Y2H [** [9]**].** (TXT 1 MB)

Additional file 3: **Text file contains the predicted interactions.** (TXT 7 MB)

Additional file 4: **Text file contains the biological processes modules that all the 9 HTLV viruses are involved in.** (TXT 2 MB)

Additional file 5: **Text file contains the molecular functional modules that all the 9 HTLV viruses are involved in.** (TXT 2 MB)

Additional file 6: **Text file contains the cellular compartments modules that all the 9 HTLV viruses are involved in.** (TXT 938 KB)

Additional file 7: **Text file contains the biological processes modules that NOT all the 9 HTLV viruses are involved in.** (TXT 401 KB)

Additional file 8: **Text file contains the molecular functional modules that NOT all the 9 HTLV viruses are involved in.** (TXT 111 KB)

Additional file 9: **Text file contains the cellular compartments modules that NOT all the 9 HTLV viruses are involved in.** (TXT 111 KB)

## References

[1] Wu X, Zhu L, Guo J, Zhang D, Lin K (2006). Prediction of yeast protein-protein interaction network: insights from the gene ontology and annotations. Nucleic Acids Res.

[2] DeBodt S, Proost S, Vandepoele K, Rouzé P, Peer Y (2009). Predicting protein-protein interactions in Arabidopsis thaliana through integration of orthology, gene ontology and co-expression. BMC Genomics.

[3] Shen J, Zhang J, Luo X, Zhu W, Yu K, Chen K, Li Y, Jiang H (2007). Predicting protein–protein interactions based only on sequences information. PNAS.

[4] von Mering C, Krause R, Snel B, Cornell M, Oliver SG, Fields S, Bork P (2002). Comparative assessment of large-scale datasets of protein-protein interactions. Nature.

[5] Edwards AM, Kus B, Jansen R, Greenbaum D, Greenblatt J, Gerstein M (2002). Bridging structural biology and genomics: assessing protein interaction data with known complexes. Trends Genet.

[6] Fu W, Sanders-Beer BE, Katz KS, Maglott DR, Pruitt KD, Ptak RG (2009). Human immunodeficiency virus type 1, human protein interaction database at NCBI. Nucleic Acids Res.

[7] Wuchty S (2011). Computational prediction of host-parasite protein interactions between P. falciparum and H. sapiens. PLoS ONE.

[8] Schleker S, Sun J, Raghavan B, Srnec M, Müller N, Koepfinger M, Murthy L, Zhao Z, Klein-Seetharaman J (2012). The current Salmonella-host interactome. Proteomics Clin Appl.

[9] Simonis N, Rual JF, Lemmens I, Boxus M, Hirozane-Kishikawa T, Gatot JS, Dricot A, Hao T, Vertommen D, Legros S, Daakour S, Klitgord N, Martin M, Willaert JF, Dequiedt F, Navratil V, Cusick ME, Burny A, Van Lint C, Hill DE, Tavernier J, Kettmann R, Vidal M, Twizere JC (2012). Host-pathogen interactome mapping for HTLV-1 and -2 retroviruses. Retrovirology.

[10] Tastan O, Qi Y, Carbonell J, Klein-Seetharaman J: **Prediction of interactions between HIV-1 and human proteins by information integration.** *Proceedings of the Pacific Symposium on Biocomputing (PSB-2009)*516–527. PMC3263379 19209727

[11] Qi Y, Tastan O, Carbonell JG, Klein-Seetharaman J, Weston J (2010). Semi-supervised multi-task learning for predicting interactions between HIV-1 and human proteins. Bioinformatics.

[12] Dyer M, Muralib T, Sobrala B (2011). Supervised learning and prediction of physical interactions between human and HIV proteins. Infect Genet Evol.

[13] Doolittle J, Gomez S (2010). Structural similarity-based predictions of protein interactions between HIV-1 and Homo sapiens. Virol J.

[14] Mukhopadhyay A, Maulik U, Bandyopadhyay S (2012). A novel biclustering approach to association rule mining for predicting HIV-1–human protein interactions. PLoS ONE.

[15] Dyer M, Murali T, Sobral B (2007). Computational prediction of host-pathogen protein-protein interactions. Bioinformatics.

[16] Schleker S, Garcia-Garcia J, Klein-Seetharaman J, Oliva B (2012). Prediction and comparison of Salmonella-human and Salmonella-Arabidopsis interactomes. Chem Biodivers.

[17] Kshirsagar M, Carbonell J, Judith K (2012). Techniques to cope with missing data in host–pathogen protein interaction prediction. Bioinformatics.

[18] Kshirsagar M, Carbonell J, Judith K (2013). Multitask learning for host–pathogen protein interactions. Bioinformatics.

[19] Mei S (2013). Probability weighted ensemble transfer learning for predicting interactions between HIV-1 and human proteins. PLoS ONE.

[20] Yu J, Guo M, Needham CJ, Huang Y, Cai L, Westhead DR (2010). Simple sequence-based kernels do not predict protein-protein interactions. Bioinformatics.

[21] Venkatesan K, Rual JF, Vazquez A, Stelzl U, Lemmens I, Hirozane-Kishikawa T, Hao T, Zenkner M, Xin X, Goh KI, Yildirim MA, Simonis N, Heinzmann K, Gebreab F, Sahalie JM, Cevik S, Simon C, de Smet AS, Dann E, Smolyar A, Vinayagam A, Yu H, Szeto D, Borick H, Dricot A, Klitgord N, Murray RR, Lin C, Lalowski M, Timm J (2009). An empirical framework for binary interactome mapping. Nat Methods.

[22] Rual JF, Venkatesan K, Hao T, Hirozane-Kishikawa T, Dricot A, Li N, Berriz GF, Gibbons FD, Dreze M, Ayivi-Guedehoussou N, Klitgord N, Simon C, Boxem M, Milstein S, Rosenberg J, Goldberg DS, Zhang LV, Wong SL, Franklin G, Li S, Albala JS, Lim J, Fraughton C, Llamosas E, Cevik S, Bex C, Lamesch P, Sikorski RS, Vandenhaute J, Zoghbi HY (2005). Towards a proteome scale map of the human protein-protein interaction network. Nature.

[23] Chatr-aryamontri A, Ceol A, Peluso D, Nardozza A, Panni S, Sacco F, Tinti M, Smolyar A, Castagnoli L, Vidal M, Cusick ME, Cesareni G (2009). VirusMINT: a viral protein interaction database. Nucleic Acids Res.

[24] Navratil V, de Chassey B, Meyniel L, Delmotte S, Gautier C, André P, Lotteau V, Rabourdin-Combe C (2009). VirHostNet: a knowledge base for the management and the analysis of proteome-wide virus-host interaction networks. Nucleic Acids Res.

[25] Freund Y, Schapire RE (1997). A decision-theoretic generalization of on-line learning and an application to boosting. J Comput Syst Sci.

[26] Vezhnevets A, Vezhnevets V (2005). Modest AdaBoost – Teaching AdaBoost to Generalize Better. Graphicon.

[27] Boeckmann B, Bairoch A, Apweiler R, Blatter MC, Estreicher A, Gasteiger E, Martin MJ, Michoud K, O'Donovan C, Phan I, Pilbout S, Schneider M (2003). The SWISS-PROT protein knowledgebase and its supplement TrEMBL. Nucleic Acids Res.

[28] Altschul S, Madden T, Schaffer A, Zhang J, Zhang Z, Miller W, Lipman D (1997). Gapped BLAST and PSI-BLAST: a new generation of protein database search programs. Nucleic Acids Res.

[29] Barrell D, Dimmer E, Huntley RP, Binns D, O'Donovan C, Apweiler R (2009). The GOA database in 2009—an integrated gene ontology annotation resource. Nucleic Acids Res.

[30] Meir R, Ratsch G (2003). An introduction to boosting and leveraging. Lect Notes Artif Int.

