# Supplementary material for: Computational reconstruction of proteome-wide protein interaction networks between HTLV retroviruses and Homo sapiens
Source: BMC Bioinformatics. 2014 Jul 18;15(1):245. doi: 10.1186/1471-2105-15-245 (PMC4133621; doi:10.1186/1471-2105-15-245)

1. Brief description of Modest AdaBoost [26].

---

1. Given training instances $(x_1, y_1),...,(x_N, y_N)$, initialize instance weights $D_0(i) = 1/N$;
2. For m = 1,…,*M* and while $f_m \neq 0$

   a. Use distribution $D_m(i)$ and weighted least squares to train weak classifier:

$$h_m(x) = \arg\min_h \left(\sum_{i=1}^{N} D_m(i) \bullet (y_i - h(x_i))^2 \right) \tag{1}$$

   b. Compute the *inverted* distribution

$$\overline{D_m(i)} = (1 - D_m(i))\overline{Z_m} \tag{2}$$

   c. Compute

$$P_m^{+1}(x) = P_{D_m}(y = +1 \wedge h_m(x)); \overline{P}_m^{+1}(x) = P_{\overline{D_m}}(y = +1 \wedge h_m(x)) \tag{3}$$

$$P_m^{-1}(x) = P_{D_m}(y = -1 \wedge h_m(x)); \overline{P}_m^{-1}(x) = P_{\overline{D_m}}(y = -1 \wedge h_m(x)) \tag{4}$$

   d. Set

$$f_m(x) = (P_m^{+1}(1 - \overline{P}_m^{+1}) - P_m^{-1}(1 - \overline{P}_m^{-1}))(x) \tag{5}$$

   Update the weight distributions

$$D_{m+1}(i) = {D_m(i)\exp(-y_i f_m(x_i))} \big/ {Z_m} \tag{6}$$

3. Construct the final classifier

$$\mathbb{F}(x) = \sum_{m=1}^{M} f_m(x) \tag{7}$$

---

where $\overline{Z_m}, Z_m$ are normalization factors, P_m^{+1}, P_m^{-1} denote the probability that the weak classifier $h_m(x)$ assigns label $\{+1, -1\}$ to the instance x under the weight distribution D_m, and similarly $\overline{P}_m^{+1}, \overline{P}_m^{-1}$ denote the probability under the inverted weight distribution $\overline{D_m}$. From formula (6), we can see that the weight increases ($D_{m+1}(i) > D_m(i)$) for those misclassified instances ($y_i f_m(x_i) < 0$). The inverted weight distribution $\overline{D_m}$ conversely assigns higher weights to those correctly-classified instances. $\overline{D_m}$ pays more attention to those hard-to-classify or noisy instances, and D_m pays more attention to those regular or noise-free instances. The hypothesis yielded in each round of training $f_m(x)$ takes both $\overline{D_m}$ and D_m into account (see formula (5)), and then all the hypothesises are finally combined into a decision committee (see formula (7)).

2. Formal definitions of SP, SE, MCC, overall accuracy (Acc) and F1 score.

$$p_l = M_{l,l}, q_l = \sum_{i=1,i\neq l}^{L} \sum_{j=1,j\neq l}^{L} M_{i,j}, r_l = \sum_{i=1,i\neq l}^{L} M_{i,l}, s_l = \sum_{j=1,j\neq l}^{L} M_{l,j}$$

$$p = \sum_{l=1}^{L} p_l, q = \sum_{l=1}^{L} q_l, r = \sum_{l=1}^{L} r_l, s = \sum_{l=1}^{L} s_l \quad (8)$$

$$SP_l = p_l / (p_l + r_l), l = 1, 2..., L$$

$$SE_l = p_l / (p_l + s_l), l = 1, 2..., L \quad (9)$$

$$MCC_l = (p_l q_l - r_l s_l) / \sqrt{(p_l + r_l)(p_l + s_l)(q_l + r_l)(q_l + s_l)}, l = 1, 2..., L$$

$$Acc = \sum_{l=1}^{L} M_{l,l} / \sum_{i=1}^{L} \sum_{j=1}^{L} M_{i,j} \quad (10)$$

$$MCC = (pq - rs) / \sqrt{(p+r)(p+s)(q+r)(q+s)}$$

where the confusion matrix $M_{i,j}$ records the counts that class i are classified to class j and L denotes the number of labels.

$$F1\_score = 2 \times SP_l \times SE_l / (SP_l + SE_l), l = 1 \text{ denotes the positive class} \quad (11)$$

3. Figure S1 ROC curve for three experimental settings (Multi-instance AdaBoost, Multi-instance AdaBoost Novel, Single-instance AdaBoost) on the dataset *S*

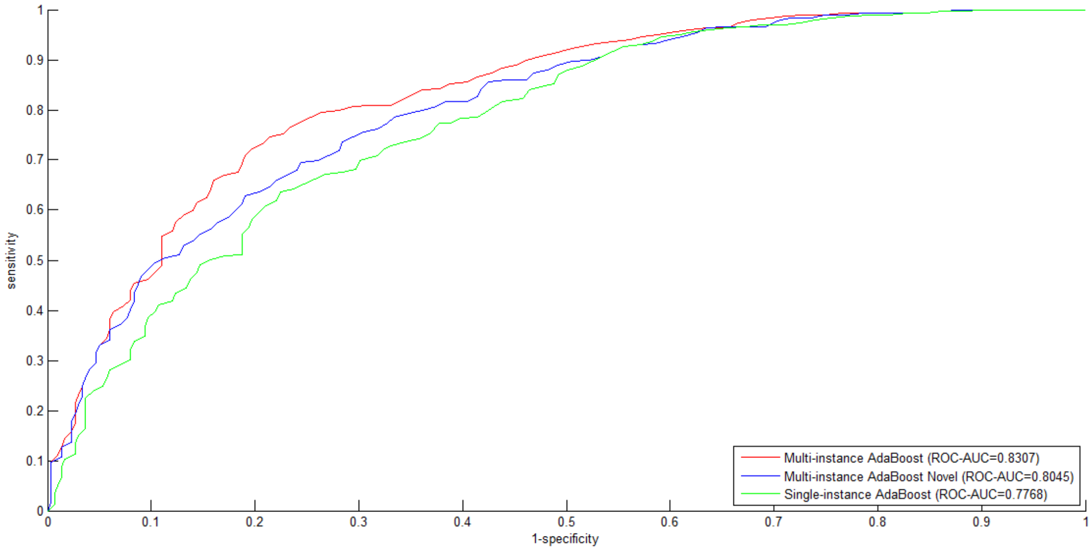

4. **Figure S2 Precision-Recall curve for three experimental settings (Multi-instance AdaBoost, Multi-instance AdaBoost Novel, Single-instance AdaBoost) on the dataset *S***

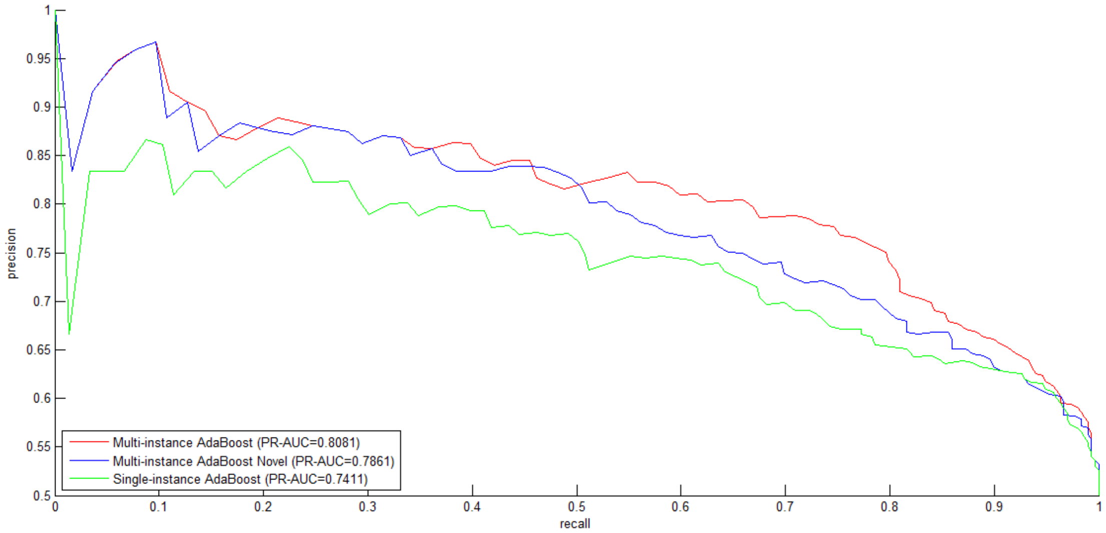

5. Table S1 10-fold cross validation performance estimation on on the dataset *S*

	Multi-instance AdaBoost			*Multi-instance AdaBoost Novel*			*Single-instance AdaBoost*		
	SP	***SE***	***MCC***	***SP***	***SE***	***MCC***	***SP***	***SE***	***MCC***
Positive (interaction)	0.7393	0.8060	0.6066	0.7129	0.7559	0.5541	0.7128	0.6722	0.5088
Negative (non-interaction)	0.7868	0.7157	0.5949	0.7402	0.6957	0.5445	0.6899	0.7291	0.5191
**[*Acc; MCC*]**	[76.09%; 0.5991]			[72.58%; 0.5486]			[70.07%;0.5134]		
**[*ROC-AUC; PR-AUC*]**	[0.8307; 0.8081]			[0.8045; 0.7861]			[0.7768; 0.7411]		
F1 Score	0.77			0.73			0.69		

6. Figure S3 The predicted HTLV-human PPI sub-network GO:0006120 *(biological process: mitochondrial electron transport, NADH to ubiquinone*). The green node denotes HTLV protein and the red node denotes human protein.

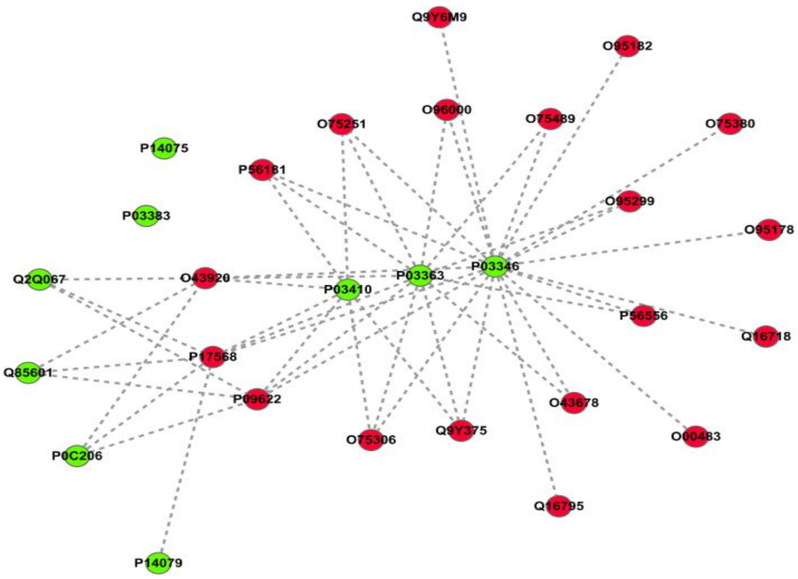

7. Figure S4 The predicted HTLV-human PPI sub-network GO:0005344 (*molecular function: oxygen transporter activity*). The green node denotes HTLV protein and the red node denotes human protein.

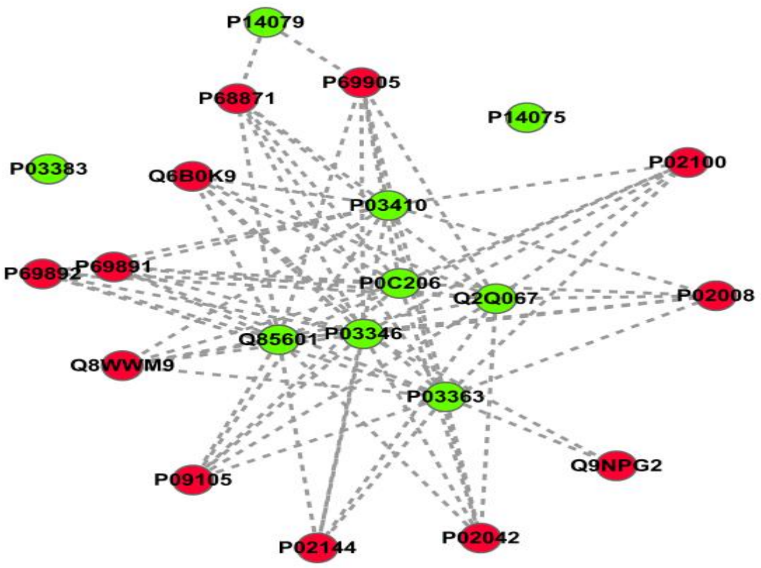

Supplement: Supplementary file 1 — Additional file 1: Brief description of Modest AdaBoost [ [26]]. Formal definitions of SP, SE, MCC, overall accuracy (Acc) and F1 score. Figure S1 ROC curve for three experimental settings (Multi-instance AdaBoost, Multi-instance AdaBoost Novel, Single-instance AdaBoost) on the dataset S. Figure S2 Precision-Recall curve for three experimental settings (Multi-instance AdaBoost, Multi-instance AdaBoost Novel, Single-instance AdaBoost) on the dataset S. Table S1 10-fold cross validation performance estimation on on the dataset S. Figure S3 The predicted HTLV-human PPI sub-network GO:0006120 (biological process: mitochondrial electron transport, NADH to ubiquinone). The green node denotes HTLV protein and the red node denotes human protein. Figure S4 The predicted HTLV-human PPI sub-network GO:0005344 (molecular function: oxygen transporter activity). The green node denotes HTLV protein and the red node denotes human protein. (PDF 139 KB) [file 12859_2014_6529_MOESM1_ESM.pdf]
